# Integration of priority population, health and nutrition interventions into health systems: systematic review

**DOI:** 10.1186/1471-2458-11-780

**Published:** 2011-10-10

**Authors:** Rifat Atun, Thyra E de Jongh, Federica V Secci, Kelechi Ohiri, Olusoji Adeyi, Josip Car

**Affiliations:** 1 Imperial College Business School, South Kensington Campus, London SW7 2AZ, UK; 2 Human Development Network, The World Bank, 1818 H St., NW, Washington DC, 20433, USA; 3 Department of Primary Care and Social Medicine, Imperial College Faculty of Medicine, London W6 8RP, UK

## Abstract

**Background:**

Objective of the study was to assess the effects of strategies to integrate targeted priority population, health and nutrition interventions into health systems on patient health outcomes and health system effectiveness and thus to compare integrated and non-integrated health programmes.

**Methods:**

Systematic review using Cochrane methodology of analysing randomised trials, controlled before-and-after and interrupted time series studies. We defined specific strategies to search PubMed, CENTRAL and the Cochrane Effective Practice and Organisation of Care Group register, considered studies published from January 1998 until September 2008, and tracked references and citations. Two reviewers independently agreed on eligibility, with an additional arbiter as needed, and extracted information on outcomes: primary (improved health, financial protection, and user satisfaction) and secondary (improved population coverage, access to health services, efficiency, and quality) using standardised, pre-piloted forms. Two reviewers in the final stage of selection jointly assessed quality of all selected studies using the GRADE criteria.

**Results:**

Of 8,274 citations identified 12 studies met inclusion criteria. Four studies compared the benefits of Integrated Management of Childhood Illnesses in Tanzania and Bangladesh, showing improved care management and higher utilisation of health facilities at no additional cost. Eight studies focused on integrated delivery of mental health and substance abuse services in the United Kingdom and United States of America. Integrated service delivery resulted in better clinical outcomes and greater reduction of substance abuse in specific sub-groups of patients, with no significant difference found overall. Quality of care, patient satisfaction, and treatment engagement were higher in integrated delivery models.

**Conclusions:**

Targeted priority population health interventions we identified led to improved health outcomes, quality of care, patient satisfaction and access to care. Limited evidence with inconsistent findings across varied interventions in different settings means no general conclusions can be drawn on the benefits or disadvantages of integrated service delivery.

## Background

Benefits of integrating targeted priority population, health and nutrition programmes into mainstream health system functions have been the subject of a longstanding debate, characterised by polarisation of views: a debate recently rekindled due to substantial increases in externally funded targeted programmes[1-3]. Furthermore, the major focus of the recent G8 summits in Japan and Italy emphasised in developing countries approaches that foster both health systems strengthening and disease-specific targeted approaches[4]. In spite of this rich debate for or against integration and how it should be achieved, however, all too frequently the arguments have not been underpinned by robust consistent evidence [1].

According to the World Health Organization (WHO), integrated health services, also called the 'horizontal' approach, represent "the process of bringing together common functions within and between organizations to solve common problems, developing a commitment to shared vision and goals and using common technologies and resources to achieve these goals"[5]. In 2008, WHO re-defined integrated health services as "organization and management of health services so that people get the care they need, when they need it, in ways that are user-friendly, achieve the desired results and provide value for money"[6]. On the other hand, targeted interventions, also called the 'vertical' approach, refer to delivery of health services focused on addressing a specific disease or a condition[7].

Our review is focused on priority population, health and nutrition programmes and their integration. These programmes, which include reproductive health, maternal and child health, communicable diseases, immunization and malnutrition,[8] represent a set of health strategies fundamental for economic and human development and poverty alleviation as set for example in the Millennium Development Goals (MDGs)[9].

Health related MDGs do not include mental disorders and mental health-related conditions (such as anxiety disorders, alcohol and drug abuse) even though they represent an important cause of sickness and disability in both developed and developing countries:[10] accounting for 40% of primary care consultations in developed countries. Their integration has been highly recommended, with the assumption that integration offers the possibility of simultaneous treatment of both mental and physical health needs[11].

The presence of both integrated and non-integrated programmes in many countries suggests there may be benefits to either approach, but the relative merits of integration, in terms of improved health outcomes, equity or efficiency, in various contexts and for different interventions have not been systematically analysed and documented[1]. Such an analysis is complex as integration is used to describe a variety of organisational arrangements in relation to key health system functions[12]. Furthermore, as the nature and extent of integration of targeted interventions into mainstream health system functions vary, there are methodological challenges to comparing various interventions.

We conducted a systematic review to assess the effects of strategies to integrate targeted priority population, health and nutrition interventions into mainstream health system delivery on patient health outcomes and effectiveness of health systems and thus compared integrated and non-integrated health programmes.

## Methods

We followed the Cochrane methodology for conducting systematic reviews[13]. Study designs considered for inclusion comprised randomised controlled trials (RCT), controlled clinical trials (CCT), interrupted time series (ITS), and controlled before and after studies (CBA). Our search algorithm was designed to include a wide range of health interventions and study settings, with no limit on type of study participants.

We included interventions focused on improving integration of priority population, health and nutrition programmes. Interventions focused on health care integration improvement refer to changes in organisation, management, planning and decision making in health care resulting in delivery of a range of services at a particular service delivery point, in provision of preventive and curative health care to a particular group of patients and in continuity of health care over time. These interventions were confined to priority population, health and nutrition programmes, i.e. reproductive health, maternal and child health, communicable diseases, immunization, malnutrition, mental health disorders and substance abuse.

We carefully defined, with the help of an information specialist, the databases that would likely yield relevant studies and specific strategies to search PubMed, the Cochrane Central Register of Controlled Trials (CENTRAL), the Cochrane Effective Practice and Organisation of Care Group (EPOC) register and Database of Abstracts of Reviews of Effectiveness.

The search strategy is detailed in Additional file 1. We also screened reference lists of the included studies and citations i.e. all references that cited any of the included studies identified using the ISI Science and Social Science Citation Index. We searched for studies published from January 1998 until September 2008. The search was limited to articles in English.

Given the wide variety of countries and care settings considered in this review, we anticipated substantial heterogeneity in utilised outcome measures and hence included in the review all outcome measures of interest. Primary outcome measures of interest were changes in health status (for instance changes in incidence, prevalence, mortality and morbidity rates or composite indices), financial protection, and user satisfaction. Secondary outcome measures included population coverage, equity, efficiency (for example changes in cost, cost-effectiveness), and quality (for example, adherence to guidelines for prevention, treatment and care).

Two reviewers (TdJ, FVS) independently performed the initial selection of studies by scanning the titles of all the retrieved references against inclusion and exclusion criteria based on relevance and scope of study (health, nutrition, population interventions; details available from authors on request). Each reviewer independently assessed 60% of the titles, with a high degree of inter-rater agreement as measured by Cohen's κ coefficient of 0.78. Both reviewers subsequently assessed for relevance all abstracts independently that passed the first phase. In case of disagreement between the reviewers the study was retained for further screening. Both reviewers then assessed full text articles of all potentially relevant studies. A third senior reviewer resolved disagreements (RA). Two reviewers (TdJ, FVS) in the final stage of selection jointly assessed quality of all selected studies using the Cochrane EPOC group criteria. A third reviewer (RA) then appraised the selected studies and confirmed their suitability for inclusion. Only studies that presented low or moderate risk of bias were included in the review.

We extracted data concerning the details of study characteristics (design, quality, randomisation, allocation), setting, intervention, participants, and outcomes (primary and secondary) using a purposely-designed data extraction sheet.

We divided the studies into two main sets based on type of intervention and care setting. Within each set we collected and compared all primary and secondary outcomes of interest. Due to the high level of heterogeneity of the evidence both within and between sets, no meta-analysis of results could be performed. We assessed the strength of the evidence for each type of outcome for the consistency of findings across studies, the directness of the evidence and the possible impact of confounding variables using the GRADE quality criteria[14,15].

## Results

### Description of the studies

**Figure 1:**
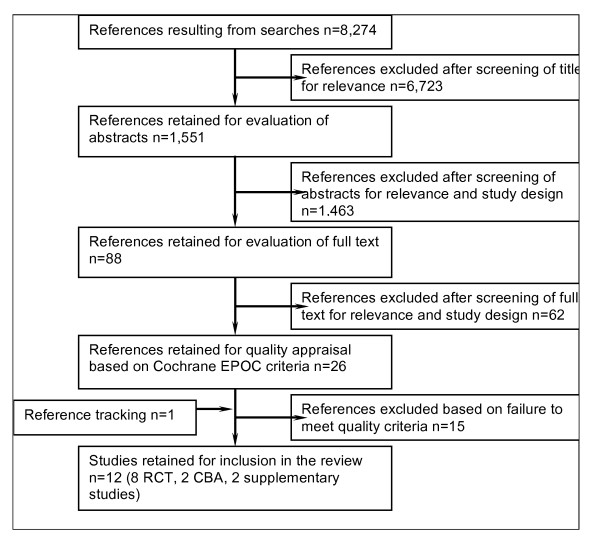
**Flow chart of the study selection process**.

We retrieved from the database search 8,274 potentially relevant articles (Figure 1). After screening titles for relevance we selected 1,551 titles for the next stage. Screening of abstracts by both reviewers for relevance and study design reduced the selection to 88 potentially suitable studies (Cohen's κ coefficient for inter-rater agreement 0.78). An analysis of the full text resulted in the exclusion of 62 more studies. We retrieved and included one additional study by reference tracking. After quality appraisal ten studies met the inclusion criteria. Five of the included studies presented low risk of bias and five moderate risk of bias (Additional file 2). Eight studies were randomised controlled trials,[16-22] and two were controlled before and after studies[23,24]. We included two additional studies as supplementary data to one of the included controlled before and after studies (Armstrong Schellenberg 2004) and relied on the same data set (Additional file 3)[23,25,26].

### Geographic location of the studies

Four of the ten studies examined implementation of the Integrated Management of Childhood Illnesses (IMCI) strategy in developing countries,[17,23,25,26] and the remaining eight studies analysed provision of mental health and/or substance abuse services in developed countries, notably the United States of America (USA) and the United Kingdom (UK). Within the latter group there were three distinct series of studies (Table 1): the first refers to the PRISM-E project in the USA in which integrated delivery of mental health or substance abuse services for elderly veterans in a primary care setting was piloted;[16,19,20] the second, from the US, focused on integration of substance abuse treatment and medical care services for patients with addiction problems and associated co-morbidities;[21,22] and the third group of studies from the US and the UK analysed the impact of integrating treatment of schizophrenia and depression respectively into primary health care[18,24,27].

**Table 1:** Classification of included studies

Programme classification	Studies	Description of intervention
Integrated Management of Childhood Illnesses (IMCI)	El Arifeen 2004 Armstrong Schellenberg 2004	Integration based on treatment guidelines and training for management of childhood illnesses

The Primary Care Research in Substance Abuse and Mental Health for the Elderly study (PRISM-E)	Bartels 2004 Krahn 2006 Oslin 2006	Integrated delivery of mental health and/or substance abuse services for elderly veterans in a primary care setting

Services for substance abuse and primary medical care	Weisner 2001 Willenbring 1999	Substance abuse treatment integrated with medical treatment of substance abuse-related co-morbidities

Mental health services in primary health care	Gater 1997 Watts 2007 Druss 2001	Treatment services for depression, schizophrenia and other mental illness integrated into primary health care

### Types of interventions

**Figure 2:**
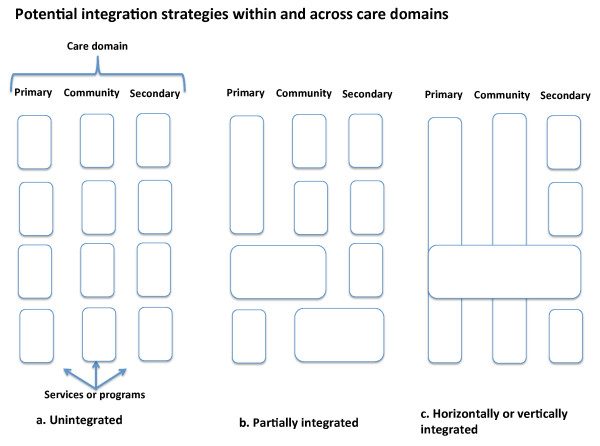
**Conceptual diagram of the different models of integration reviewed in the study**.

We distinguished two main groups of studies. The first group compared IMCI with 'routine care' comprising separate distinct programmes for managing childhood illness[17,23,25,26]. The second group compared mental health and/or substance abuse treatment services integrated into primary health care or with specialist community teams with 'routine' mental health services including those provided in hospitals, and substance abuse treatments delivered as stand alone specialist services with no integration to primary health care or other services delivered in the community[16,18-22,24,27]. Figure 2 illustrates conceptually potential integration strategies within and across primary, community and secondary care domains.

### Findings from the studies

#### Integrated Management of Childhood Illnesses

IMCI is a systematic approach to children's health, which focuses on the child as a whole, rather than on a single disease or condition. The approach, developed by WHO and UNICEF, aims to reduce death, illness and disability, and promote improved growth and development among children under five years of age. Its implementation emphasises the use of clinical guidelines adapted to a country context. The IMCI strategy stresses coordinated activities within three components aimed at improving: (1) the performance of health workers in the prevention and treatment of childhood diseases; (2) the organisation and operation of health services so they provide quality care; and (3) family and community care practices (e.g. appropriate care seeking behaviour or improved nutrition). IMCI focuses on both prevention and treatment implemented by families and communities as well as by healthcare providers. Children brought for medical treatment in the developing world are often suffering from more than one condition, making a single diagnosis difficult. IMCI ensures the combined treatment of the major childhood illnesses, emphasizing prevention of disease through immunization and improved nutrition.

In Tanzania, Schellenberg et al. (2004) conducted a controlled before-and-after study to compare child health and survival as well as economic costs and benefits between two rural districts where IMCI had been implemented for two years prior to the evaluation with two neighbouring control districts that used routine care comprising disease-specific approaches but not IMCI[23]. The study districts had comparable geographic, demographic and mortality profiles at the time of introduction of IMCI.

In the intervention districts, case management improved for a number of measures for correct diagnosis and treatment (Table 2). Furthermore, the under-5 mortality rate was 13% lower in IMCI districts than in comparison areas. Prevention behaviours, such as use of mosquito nets, all favoured the comparison districts. Care seeking behaviour appeared unaffected under IMCI, whereas knowledge of caregivers on correct use of oral rehydration salts significantly improved. Two related studies found that implementation of IMCI was not more costly than routine care,[25] and led to significant improvements in case management at costs similar to or lower than those of conventional case-management[26].

**Table 2:** Outcomes for studies on IMCI

Type of outcome	Study	Measure	Outcome (IMCI vs. Control area)
**Health outcomes**	Armstrong Schellenberg 2005	Death rate per 1000 child years.	From 27.2 to 24.4 vs. from 27.0 to 28.2, (p = 0.28)
		Carer of child prescribed oral medication reports correctly how to give treatment.	163/225 (72%) vs. 100/179 (56%), (p = 0.02)

**Quality of care**	Armstrong Schellenberg 2005	Children checked for presence of cough, diarrhoea and fever.	219/231 (95%) vs. 67/188 (36%), (p < 0.0001)
		Children correctly classified.	139/219 (63%) vs. 66/176 (38%), (p < 0.0001)
		Correct prescription of oral antibiotics and/or oral anti-malarials.	159/219 (73%) vs. 63/178 (35%), (p < 0.0001)
	
	El-Arifeen 2004	Mean index of correct treatment and counselling.	From 8 to 54 vs. from 5 to 9, (p < 0.001)

**Utilisation of services**	Armstrong Schellenberg 2005	Change in appropriate care seeking behaviour.	From 211/512 (41%) to 203/531 (38%) vs. from 209/502 (42%) to 138/427 (30%), (p = 0.45)
	
	El-Arifeen 2004	Ill children taken to a health facility or health worker.	From 10% to 19% vs. from 6% to 9%

**Cost**	Adam 2005	Annualised cost of care.	US$ 11.19 vs. US$ 16.09
	
	Bryce 2005	Cost per child visit managed correctly.	US$ 4.02 vs. US$ 25.70

In Bangladesh, a cluster-randomised controlled trial compared 20 randomly selected facilities in which nationally adapted IMCI case management guidelines were introduced with 20 paired facilities that had not implemented the guidelines[17]. The quality of care (as measured by adherence to IMCI guidelines), care-seeking behaviour and utilization of governmental health facilities had all improved 18 months after introduction of IMCI.

#### Integrating substance abuse treatment for patients with substance abuse-related conditions

We found two studies focused on integration of addiction treatment programmes. Both studies compared the integrated management of substance abuse treatment and medical care of substance abuse-related co-morbidities with independent, routine treatment approaches where addiction treatment was provided separately from comprehensive medical care.

Weisner et al. (2001) conducted a randomised controlled trial in Sacramento, US, to compare the effectiveness, service utilization and treatment costs of a substance abuse programme in which primary health care services were integrated within the treatment unit with a control programme in which patients received the same set of substance abuse services but where medical care was provided in separate primary care clinics[21]. The 285 patients randomly assigned to the intervention arm included 169 patients with substance abuse-related medical conditions (SAMC) and 116 patients with a substance abuse problem but no associated medical conditions (non-SAMC). The study found no differences in total, alcohol, and other drug abstinence rates for the non-SAMC patients (Table 3). However, patients with medical or psychiatric SAMCs who received integrated care had higher total and alcohol abstinence rates and longer periods of abstinence without a significant increase in average cost of all treatment per month.

**Table 3:** Outcomes for studies on integrated delivery of mental health and substance abuse services

Type of outcome	Study	Measure	Outcome (Intervention vs. Control)
**Health outcomes**	Druss 2001	Change in physical component summary index.	+4.7% vs. -0.3%, (p < 0.001)
		Change in mental component summary index.	+2.4% vs. +2%, (p = 0.84)
	
	Krahn 2006	Change in Centre for Epidemiological Studies Depression scale (CES-D) score.	Patients with all depression: -6.0 ± 12.0 vs. -7.8 ± 11.8, (p = 0.07)
			Patients with major depression: -7.5 ± 13.1 vs. -10.2 ± 12.1, (p = 0.003)
		Change in Medical Component Score (MCS).	Patients with all depression: +4.8 ± 12.6 vs. +4.9 ± 12.9, (p = 0.88)
			Patients with major depression: +5.9 ± 12.6 vs. + 6.8 ± 12.8, (p = 0.32)
	
	Willenbring 1999	Number of patients with 2-year survival.	31/38 (81%) vs. 26/37 (70%), (p = 0.03)

**Drug and alcohol use**	Oslin 2006	Change in number of drinks per week.	-6.0 vs. -5.9 (p = 0.913)
		Change in number of binge episodes in the preceding three months.	-8.5 vs. -10.2 (p = 0.750)
	
	Weisner 2001	Total abstinence and duration of abstinence.	Non-SAMC patients: 66% vs. 73%, (p = 0.23)
			SAMC patients: 69% vs. 55%, (p = 0.006); period of abstinence 135 days vs. 122 days, (p = 0.05)
		
		Alcohol abstinence.	Non-SAMC patients: 73% vs. 78% (p = 0.41)
			SAMC patients: 80% vs. 65%, (p = 0.002)
		
		Other drug abstinence	Non-SAMC patients: 84% vs. 87%, (p = 0.50)
	
	Willenbring 1999	Number of patients with alcohol abstinence after 2 years.	28/38 (74%) vs. 17/36 (48%), (p = 0.02)

**Patient satisfaction**	Druss 2001	Satisfaction score on 47-item questionnaire.	Patients in integrated model were more satisfied with overall care received in 6 of 8 domains (*access, attention to patient preferences, courtesy, coordination, continuity, and overall care*) (p < 0.05 on all 6 domains)
	
	Gater 1997	Score on Client Satisfaction Questionnaire (range 1-4; low score indicates higher satisfaction).	1.86 vs. 2.23

**Quality of care**	Druss 2001	Delivery of preventive measures outlined in clinical guidelines.	Patients in integrated model (n = 59) more likely than in control group (n = 61) to receive 15 of 17 measures, (p < 0.01)
	
	Gater 1997	Number of clinical needs met; and unmet.	2.62 vs. 1.60, (p < 0.001); 0.57 vs. 1.62 (p < 0.001)
		Number of social needs met; and unmet.	1.83 vs. 1.49, (p = NS); 0.86 vs. 1.64 (p < 0.05)
	
	Watts 2007	Patients who screened positive for depression and received treatment in accordance with guidelines.	From 1.1% to 11.2% vs. from 3.0% to 0.7%, (p < 0.001)

**Utilisation of services**	Bartels 2004	Mean number of mental health and substance abuse visits.	3.04 vs. 1.91 (p ≤ 0.001)
		Appointment attendance.	71% vs. 48.8% (95% CI = 2.14 to 3.08)
	
	Druss 2001	Patients who used a medical: Primary care service; Specialty service; Emergency department; Inpatient service.	54/59 (91.5%) vs. 44/61 (72.1%), (p = 0.006); 41/59 (69.5%) vs. 41/61 (67.2%), (p = 0.17); 7/59 (11.9%) vs. 16/61 (26.2%), (p = 0.04); 5/59 (8.5%) vs. 11/61 (18%), (p = 0.12)
		Patients who used a mental health: Outpatient service; Emergency department; Inpatient service.	58/59 (98.3%) vs. 61/61 (100%), (p = 0.31); 21/59 (35.6%) vs. 25/61 (41%), (p = 0.31); 8/59 (13.6%) vs. 10/61 (16.4%), (p = 0.66)
	
	Willenbring 1999	Mean number of IOT visits in 2 years.	42.2 ± 29.1 vs. 17.4 ± 15.6, (p < 0.001)
		Mean number of IOT visits in first and last 6 months of treatment.	From 14 to 9 vs. 4-6 in both periods

**Access to health care**	Watts 2007	Patients who screened positive and were able to access mental health services.	36.0% vs. 9%, (p < 0.001)

**Cost**	Druss 2001	Mean cost per subject treated	US$ 13,010 vs. US$ 14,543
	
	Gater 1997	Overall per capita health service cost	£ 1,406 vs. £ 1,199
	
	Weisner 2001	Average cost of all treatment per month	US$ 470.81 vs. US$ 427.95, (p = 0.14)

A randomised controlled trial by Willenbring et al. (1999) in Minneapolis, USA compared veterans (n = 48) with a diagnosis of severe alcohol-related medical illness who received Integrated Outpatient Treatment (IOT) for medical problems and alcoholism through a single referral appointment at the Minneapolis Veterans Affairs Medical Centre (MVAMC) with a control group (n = 53) that received routine care comprising outpatient medical services in the medical and specialty medicine clinics of the MVAMC[22]. The intervention group was simultaneously evaluated for alcoholism and, if needed, recommended for alcoholism treatment at an independent treatment facility. The integrated care led to higher patient engagement compared with routine care (Table 3). The average number of IOT visits for patients in the integrated model gradually decreased, in contrast to the control group where the number of visits did not change. Abstinence rates after two years were significant in both study arms, but higher in the group receiving IOT. The effect on two-year survival was statistically not significant.

#### Integrating mental health services in primary care

The Primary Care Research in Substance Abuse and Mental Health for the Elderly study (PRISM-E), a multi-site randomised controlled trial (RCT), compared an integrated model of mental health and substance abuse services in primary health care with an enhanced referral model (i.e. services in a specialised mental health/substance abuse clinic, physically separate from the primary health care unit)[16,19,20]. The study, conducted in five Department of Veteran Affairs (VA) Medical Centres, three community health centres and two outpatient hospital networks, compared service use, clinical outcomes and costs of service delivery for older people with depression, anxiety or at-risk alcohol consumption. In 9 out of 10 settings, there were more mental health and substance abuse visits and higher appointment attendance in the integrated care model for all demographic and diagnostic groups (Table 3)[16]. For patients suffering depression, depression severity declined and mental functioning improved in both models. No significant differences were found, except for patients with major depression for whom the enhanced referral model produced better symptomatic outcomes[19]. For older patients with at-risk alcohol consumption behaviour, there were reductions in both the quantity and frequency of drinking and binge drinking in both treatment modalities. However, no statistically significant differences were found on either measure between the two treatment models[20].

Watts et al. (2007) conducted a retrospective before-and-after study in Vermont (USA) to assess the quality of care and access to treatment for patients diagnosed with depression and treated in a primary mental health clinic [PMHC] at a Veteran Affairs medical centre consisting of open access mental health services co-located in a primary health care clinic but with no new staff added compared to usual treatment (comprising of community-based outreach clinics associated with primary health care and mental health clinics at the VA medical centre)[24]. The percentage of patients who screened positive for depression and received optimal treatment in accordance with guidelines increased at the intervention facility but declined at the control sites (Table 3). Substantially more patients who screened positive were able to access mental health services in the PMHC model and received treatment sooner than the community-based clinics.

An RCT was conducted by Gater et al. (1997) to assess the quality and cost of care provided to schizophrenic patients by general practitioners linked to a new specialist community team compared to general practitioners who used the usual hospital-based service[18]. Patients treated by the new community team were more satisfied with their care and reported fewer unmet needs than patients referred to specialist hospital psychiatric units (Table 3). Better quality of care was maintained in the community team model four years after its introduction. Cost differences between the two models of care were not significant.

Druss et al. (2001) used an RCT to compare a model that provided integrated medical and mental health services in a primary care setting for older patients with serious mental health disorders, to a routine care model in which primary care is provided by a VA general medicine clinic after referral from a mental health clinic[27]. The intervention was associated with greater access to primary and preventive care, and bigger improvements in service quality, user satisfaction, and health related quality of life, at no significant difference in cost (Table 3).

Analysis on the basis of the GRADE statement showed high quality of evidence that IMCI strategies reduced under-5 mortality and moderate quality of evidence that IMCI improves the quality of care. As for the mixed impact that IMCI had on utilisation of health services, we judged the quality of evidence as low (Annex 3, A).

Evidence grading on studies of integrated delivery of mental health and/or substance abuse treatment services showed that there was a moderate quality of evidence that the quality of care and patient satisfaction improved under integrated models of care and a low quality of evidence that integrated health services improved treatment engagement and access to mental health services. The quality of evidence which suggested no difference in health care outcomes and that for 2-year survival across care models was also graded as moderate (Annex 3, B).

## Discussion

Our systematic review shows that evidence on the relative benefits of integration of priority population, health and nutrition interventions (i.e. targeted versus integrated delivery of health services) is limited and too weak to allow for clear conclusions about when either approach is desirable. The limited evidence available suggests that integrated approaches to delivering health services, compared with targeted unintegrated approaches, improve outcomes in selected areas. It is critical that this important question receives the attention of researchers that it deserves and that we move beyond the experts' information, viewpoints and conceptual frameworks guiding billions of dollars invested in priority population, health and nutrition interventions.

Studies assessing the implementation of the IMCI programme in developing countries illustrated that integration based on implementation of case management guidelines improved quality and utilization of health care with no significant increase in cost delivery[17,23,25,26]. Results of eight eligible studies from developed countries (USA and UK) that analysed integration of service delivery for mental health disorders and substance abuse into community or primary health care are more complex. Nevertheless, integrated management of substance abuse treatment and substance abuse-related co-morbidities health care resulted in higher abstinence rates compared to standard treatment approaches where addiction treatment was provided separately from comprehensive medical care[21,22]. Studies evaluating integrated models of mental health in primary health care demonstrated advantages of integration[16,18-20,24,27].

The key benefits of integrated models of service delivery in this review were improved quality of care and clinical outcomes,[17,18,21-24,26,27] greater treatment engagement for patients who are resistant to treatment or difficult to reach in more conventional care models,[16,19,22,24,27] and improved patient satisfaction and targeting of resources[18,27].

It is, however, important to note that even in these studies these benefits do not always accrue to all patients treated under the integrated care model. Whereas Weisner et al. (2001) found no benefits for integrated delivery of substance abuse treatment and medical services in patients without substance abuse-related co-morbidities,[21] Krahn et al. (2006) demonstrated that the enhanced referral model produced better symptomatic outcomes for patients with major depression than the integrated model did, possibly due to better access to more specialised psychiatric and medication management services[19].

Although the quality of the evidence from the included studies was rated as moderate to high according to GRADE appraisal and demonstrated various benefits of integrated health care programmes, these findings cannot be generalized. Evaluated interventions as well as the definitions of integration of health care programmes across the studies included in our review were very heterogeneous. Furthermore, care setting, participants, interventions used as controls and types of outcomes all differed significantly and therefore do not allow us to form an overall conclusion on the effectiveness of integrated health programmes. In practice most health services combine non-integrated and integrated elements, but the balance between programmes in these elements varies considerably and is rarely clearly spelled out even in a research context. Hence, when programme designs are being researched, more clarity is needed on the programme element being referred to: e.g. governance arrangements, organisation, funding and service delivery.

## Conclusions

In contrast to the systematic review by Briggs et al. (2006) on strategies for integrating primary health services in middle- and low-income countries at the point of delivery we included in our review studies from all countries regardless of income[28]. While this increased heterogeneity it provided additional valuable information for policymakers who often have to make decisions that encompass different settings and conditions. Our review provides important new evidence from studies in developed countries but does not significantly change the findings of Briggs et al. (2006) that there are few rigorous studies exploring the relative merits of integrating or not integrating programmes that emphasize specific interventions[28].

Given the paucity of evidence, we suggest that in order to deliver an evidence-based conclusion on effectiveness of health programme integration, investments should be made in studies with robust designs, where possible comparable control and intervention groups, a clear and comprehensive definition of integration, valid and reliable outcomes and analysis of costs. These studies should be longitudinal in nature, carried out over a period of few years so that sustainability and long term impacts of horizontal approach could also be evaluated. Such studies will also need to take account of the multiple dimensions of integration, the wider health system context and the political economy in which they are set as these factors work beyond the interventions to determine the success of the programmes[12].

## Competing interests

The authors declare that they have no competing interests.

## Authors' contributions

RA conceived and led the design of the study, oversaw the review, drafted the manuscript. TEdJ and FVS undertook the search, data extraction and analysis and participated in drafting of the manuscript. KO helped draft the manuscript. OA helped to draft the manuscript. JC participated in the analysis, and synthesis of the findings and participated in drafting of the manuscript. All authors read and approved the final manuscript.

## Pre-publication history

The pre-publication history for this paper can be accessed here:

http://www.biomedcentral.com/1471-2458/11/780/prepub

## Supplementary Material

Additional file 1 **Box 1: Search strategy**. Click here for file

Additional file 2 **Description of included studies**. Click here for file

Additional file 3 **Description of additional studies with complementary data for Armstrong Schellenberg 2004 study**. Click here for file
