# Supplementary material for: Integration of priority population, health and nutrition interventions into health systems: systematic review
Source: BMC Public Health. 2011 Oct 10;11:780. doi: 10.1186/1471-2458-11-780 (PMC3204262; doi:10.1186/1471-2458-11-780)
Supplement: Additional file 1 — Box 1: Search strategy. [file 1471-2458-11-780-S1.DOC]

**Box 1: Search strategy**

We developed a search strategy based on the use of –exploded– MeSH terms, supplemented with a

broad search for keywords in the titles or abstracts for which no appropriate MeSH terms existed. An

earlier systematic review by Briggs and Garner that used Cochrane Systematic Review methodology

and explored integration of primary health services in middle- and low-income countries at the point of

delivery served as the basis for the development of the search strategy.[18] The strategy combines

two parts: the first designed to identify articles related to organizational arrangements for health care

delivery and the second designed to limit the search to specific areas of interest. The search strategy

is shown below.

1. ((vertical[Tiab] OR horizontal[Tiab] OR integrat*[Tiab] OR coordinat*[Tiab] OR co-ordinat*[Tiab] OR

link*[Tiab]) AND (program*[Tiab] OR care[Tiab] OR service*[Tiab]) OR "Delivery of Health Care,

Integrated"[Mesh])

AND

2. ((“child health services”[MeSH] NOT “early intervention (education)”[MeSH]) OR “immunization

programmes” [MeSH] OR “family planning services”[MeSH] OR (“maternal health services”[MeSH]

NOT “preconception care”[MeSH]) OR “maternal-child health centres”[MeSH] OR “community health

centres”[MeSH] OR immunization[MeSH] OR “reproductive medicine”[MeSH] OR “adolescent health

services”[MeSH] OR cholera[MeSH] OR dengue[MeSH] OR “Dengue Hemorrhagic Fever”[MeSH] OR

Fascioliasis[MeSH] OR Trypanosomiasis[MeSH] OR Leishmaniasis[MeSH] OR Elephantiasis[MeSH]

OR yaws[Mesh] OR “buruli ulcer”[MeSH] OR dracunculiasis[MeSH] OR leprosy[MeSH] OR

schistosomiasis[MeSH] OR helminths[MeSH] OR “chagas disease”[MeSH] OR onchocerciasis[MeSH]

OR “alcohol-related disorders”[MeSH] OR “cocaine-related disorders”[MeSH] OR “opioid-related

disorders”[MeSH] OR “substance abuse, intravenous”[MeSH] OR “anxiety disorders”[MeSH] OR

“depressive disorder”[MeSH] OR schizophrenia[MeSH] OR “eating disorders”[MeSH] OR

dementia[MeSH] OR HIV[MeSH] OR tuberculosis[MeSH] OR malaria[MeSH] OR “sexually

transmitted diseases”[MeSH] OR “mental health”[MeSH] OR “Anaemia, Iron-Deficiency”[MeSH] OR

“Vitamin A Deficiency”[MeSH] OR “Food, Fortified”[MeSH] OR micronutrients[MeSH] OR zinc[MeSH])
