# Supplementary material for: Integration of priority population, health and nutrition interventions into health systems: systematic review
Source: BMC Public Health. 2011 Oct 10;11:780. doi: 10.1186/1471-2458-11-780 (PMC3204262; doi:10.1186/1471-2458-11-780)
Supplement: Additional file 2 — Description of included studies. [file 1471-2458-11-780-S2.PDF]

Additional file 1

Description of included studies

**Study**	**El-Arifeen 2004**
Design	Randomised Controlled Trial
Participants	20 randomly selected health facilities in an area not covered by child and reproductive health services provided by the ICDDR,B Centre for Health and Population compared to 20 paired control facilities
Interventions	IMCI with case management guidelines adapted for Bangladesh
Outcomes	i) Quality of care (adherence to IMCI guidelines); ii) Care seeking behaviour; iii) Utilization of governmental health facilities
Country	Bangladesh
Notes	IMCI
Allocation concealment	Yes
Study quality	A = Low Risk of Bias

**Study**	**Bartels 2004**
Design	Randomised Controlled Trial
Participants	Older veterans with mental health or substance abuse problems
Interventions	Integrated Mental Health or Substance Abuse services in the PHC clinic by a mental health provider compared to an enhanced referral model
Outcomes	Treatment engagement defined as: i) Attendance at an appointment with a Mental Health / Substance Abuse provider following randomization at the index primary care visit, ii) Number of visits
Country	USA
Notes	PRISM-E
Allocation concealment	Not clear
Study quality	B = Moderate Risk of Bias

**Study**	**Druss 2001**
Design	Randomised Controlled Trial
Participants	Older veterans with mental health problems
Interventions	Medical care provided in an integrated setting, compared with referral to a VA general medicine clinic
Outcomes	i) Service use; ii) Quality of care; iii) Patient satisfaction; iv) Physical and mental health status; v) Costs
Country	USA
Notes	
Allocation concealment	Not clear
Study quality	B = Moderate Risk of Bias

**Study**	**Gater 1997**
Design	Randomised Controlled Trial
Participants	Patients presenting at GP practices with mental health problems
Interventions	Mental health care provided by a new community team based closely linked to PHC units, compared with traditional hospital-based services
Outcomes	i) Met and unmet needs of care using standardised instruments; ii) *Per capita* cost of services per patient; iii) Patient satisfaction

Country	United Kingdom
Notes	
Allocation concealment	Not clear
Study quality	B = Moderate Risk of Bias

**Study**	**Krahn 2006**
Design	Randomised Controlled Trial
Participants	Older patients with depression at a veterans clinic
Interventions	Integrated mental health and/or substance abuse services in the PHC clinic by a mental health provider compared with an enhanced referral model where services were provided in a physically separate specialty setting
Outcomes	i) Severity of depressive symptoms (CES-D scale) and mental functioning (MCS, SF-36); ii) remission and categorical presence or absence of depressive syndromes (MINI), and presence or absence of a 50% decrease in depressive symptoms on the CES-D. Outcomes were measured at baseline and follow-up at 3 and 6 months
Country	USA
Notes	PRISM-E
Allocation concealment	Yes
Study quality	A = Low Risk of Bias

**Study**	**Oslin 2006**
Design	Randomised Controlled Trial
Participants	Older patients with at-risk alcohol consumption at a veterans clinic
Interventions	Integrated alcohol abuse services in the PHC clinic by a mental health provider compared with an enhanced referral model where services were provided in a physically separate specialty setting
Outcomes	i) Quantity and frequency of alcohol use seven days before each assessment; ii) Number of binge dinking episodes 3 months before each assessment. Outcomes were measured at baseline and follow-up at 3 and 6 months
Country	USA
Notes	PRISM-E
Allocation concealment	Yes
Study quality	A = Low Risk of Bias

**Study**	**Armstrong Schellenberg 2004**
Design	Controlled Before-and-After Trial
Participants	4 rural districts; 2 intervention districts having received IMCI for 2 years before the study was conducted and 2 control districts with routine case management
Interventions	Integrated Management of Childhood Illness
Outcomes	i) Quality of care measured as: a) Percentage of children checked for presence of cough, diarrhoea and fever; b) Percentage of children correctly classified; c) Correct prescription of oral antibiotics and/or oral antimalarials; ii) Knowledge and care seeking behaviour of care givers; iii) Under-5 mortality rate; iv) Economic cost
Country	Tanzania
Notes	IMCI; see also Adam 2005 & Bryce2005
Allocation concealment	Yes
Study quality	A = Low Risk of Bias

**Study**	**Watts 2007**
Design	Controlled Before-and-After Trial
Participants	Patients screened positive for depression at a veterans clinic
Interventions	Mental health services provided at a Primary Mental Health Clinic (PMHC) by MH workers co-located in the PHC clinic, and working collaboratively with PHC staff; advanced or open access to MH services; and use of common standard assessment instruments
Outcomes	i) Quality of depression treatment based on adherence to guidelines; ii) Access to mental health care based on number of depressive patients seen and length of waiting time
Country	USA
Notes	
Allocation concealment	Yes
Study quality	B = Moderate Risk of Bias

**Study**	**Weisner 2001**
Design	Randomised Controlled Trial
Participants	Patients meeting criteria for alcohol or other drug abuse or dependence
Interventions	Integrated services where primary health care is provided along with substance abuse treatment within the unit
Outcomes	i) Addiction severity measured as alcohol and drug abstinence at follow-up; ii) Health care utilization; iii) Treatment costs
Country	USA
Notes	
Allocation concealment	Not clear
Study quality	B = Moderate Risk of Bias

**Study**	**Willenbring 1999**
Design	Randomised Controlled Trial
Participants	Male veterans with alcoholic dependence and severe medical co-morbid conditions
Interventions	Integrated treatment for medical problems and alcoholism through a single referral appointment
Outcomes	i) Drinking behaviour and abstinence; ii) Health service utilization (outpatient and hospital visits); iii) Self-reported well-being; iv) 2-year survival
Country	USA
Notes	
Allocation concealment	Yes
Study quality	A = Low Risk of Bias

Annex 2 Description of additional studies with complementary data for Armstrong Schellenberg 2004 study

**Study**	**Adam 2005**
Design	Cost Effectiveness Analysis
Participants	4 rural districts; 2 intervention districts having received IMCI for 2 years before the study was conducted and 2 control districts with routine case management
Interventions	Integrated Management of Childhood Illness
Outcomes	i) Total economic costs of start up and implementation of IMCI in a district; ii) Incremental costs of introducing and running IMCI
Country	Tanzania
Notes	IMCI; complementary to Armstrong Schellenberg 2004
Allocation concealment	Not applicable
Study quality	Not applicable

**Study**	**Bryce2005**
Design	Non-randomised Controlled Trial
Participants	4 rural districts; 2 intervention districts having received IMCI for 2 years before the study was conducted and 2 control districts with routine case management
Interventions	Integrated Management of Childhood Illness
Outcomes	i) Costs at national, district, facility and household levels; ii) Quality of care using a newly developed composite measure for 'correct management of childhood illness' defined as the proportion of children managed correctly for all presenting conditions and without inappropriate prescribing of antibiotics or anti-diarrhoeals
Country	Tanzania
Notes	IMCI; complementary to Armstrong Schellenberg 2004
Allocation concealment	Not applicable
Study quality	Not applicable
