# Supplementary material for: Integration of priority population, health and nutrition interventions into health systems: systematic review
Source: BMC Public Health. 2011 Oct 10;11:780. doi: 10.1186/1471-2458-11-780 (PMC3204262; doi:10.1186/1471-2458-11-780)
Supplement: Additional file 3 — Description of additional studies with complementary data for Armstrong Schellenberg 2004 study. [file 1471-2458-11-780-S3.PDF]

Additional file 2

Description of additional studies with complementary data for Armstrong Schellenberg 2004 study

**Study**	**Adam 2005**
Design	Cost Effectiveness Analysis
Participants	4 rural districts; 2 intervention districts having received IMCI for 2 years before the study was conducted and 2 control districts with routine case management
Interventions	Integrated Management of Childhood Illness
Outcomes	i) Total economic costs of start up and implementation of IMCI in a district; ii) Incremental costs of introducing and running IMCI
Country	Tanzania
Notes	IMCI; complementary to Armstrong Schellenberg 2004
Allocation concealment	Not applicable
Study quality	Not applicable

**Study**	**Bryce2005**
Design	Non-randomised Controlled Trial
Participants	4 rural districts; 2 intervention districts having received IMCI for 2 years before the study was conducted and 2 control districts with routine case management
Interventions	Integrated Management of Childhood Illness
Outcomes	i) Costs at national, district, facility and household levels; ii) Quality of care using a newly developed composite measure for 'correct management of childhood illness' defined as the proportion of children managed correctly for all presenting conditions and without inappropriate prescribing of antibiotics or anti-diarrhoeals
Country	Tanzania
Notes	IMCI; complementary to Armstrong Schellenberg 2004
Allocation concealment	Not applicable
Study quality	Not applicable
